# 6-(2-Methyl­prop­yl)-4-oxo-2-sulfanyl­idene-1,2,3,4-tetra­hydro­pyrimidine-5-carbonitrile

**DOI:** 10.1107/S1600536812005119

**Published:** 2012-02-10

**Authors:** Omar A. Al-Deeb, Ali A. El-Emam, Abdulghafoor A. Al-Turkistani, Seik Weng Ng, Edward R. T. Tiekink

**Affiliations:** a Department of Pharmaceutical Chemistry, College of Pharmacy, King Saud University, Riyadh 11451, Saudi Arabia; b Department of Chemistry, University of Malaya, 50603 Kuala Lumpur, Malaysia; c Chemistry Department, Faculty of Science, King Abdulaziz University, PO Box 80203 Jeddah, Saudi Arabia

## Abstract

The title thio­uracil derivative, C_9_H_11_N_3_OS, exists in the thione form. The six atoms comprising the ring are almost coplanar [r.m.s. deviation = 0.015 Å] and the 2-methyl­propyl group lies approximately perpendicular to this plane [the N—C—C—C torsion angle is 72.88 (14)°]. Linear supra­molecular chains along [001] sustained by N—H⋯O and N—H⋯S hydrogen bonding feature in the crystal packing.

## Related literature

For the biological activity of uracil and pyrimidine derivatives see: Ding *et al.* (2006 ▶); Hawser *et al.*, (2006 ▶); Brunelle *et al.* (2007 ▶); Al-Safarjalani *et al.* (2005 ▶); Al-Omar *et al.* (2010 ▶); Al-Abdullah *et al.* (2011 ▶); Al-Turkistani *et al.* (2011 ▶). For related uracil structures, see: Tiekink (1989 ▶); Nasir *et al.* (2010 ▶); El-Emam *et al.* (2011 ▶).
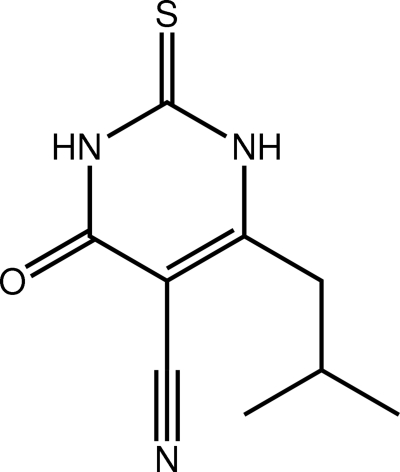


## Experimental

### 

#### Crystal data

C_9_H_11_N_3_OS
*M* *_r_* = 209.27
Monoclinic, 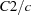

*a* = 25.8985 (6) Å
*b* = 7.0479 (2) Å
*c* = 11.1811 (2) Å
β = 98.527 (2)°
*V* = 2018.33 (8) Å^3^
*Z* = 8
Cu *K*α radiation
μ = 2.62 mm^−1^
*T* = 100 K
0.35 × 0.20 × 0.03 mm

#### Data collection

Agilent SuperNova Dual diffractometer with Atlas detector
Absorption correction: multi-scan (*CrysAlis PRO*; Agilent, 2011 ▶) *T*_min_ = 0.461, *T*_max_ = 0.926
6870 measured reflections
2102 independent reflections
1989 reflections with *I* > 2σ(*I*)
*R*_int_ = 0.024

#### Refinement

*R*[*F* ^2^ > 2σ(*F* ^2^)] = 0.030
*wR*(*F* ^2^) = 0.086
*S* = 1.03
2102 reflections
135 parameters
2 restraints
H atoms treated by a mixture of independent and constrained refinement
Δρ_max_ = 0.27 e Å^−3^
Δρ_min_ = −0.28 e Å^−3^

### 

Data collection: *CrysAlis PRO* (Agilent, 2011 ▶); cell refinement: *CrysAlis PRO*; data reduction: *CrysAlis PRO*; program(s) used to solve structure: *SHELXS97* (Sheldrick, 2008 ▶); program(s) used to refine structure: *SHELXL97* (Sheldrick, 2008 ▶); molecular graphics: *ORTEP-3* (Farrugia, 1997 ▶) and *DIAMOND* (Brandenburg, 2006 ▶); software used to prepare material for publication: *publCIF* (Westrip, 2010 ▶).

## Supplementary Material

Crystal structure: contains datablock(s) global, I. DOI: 10.1107/S1600536812005119/hg5174sup1.cif

Structure factors: contains datablock(s) I. DOI: 10.1107/S1600536812005119/hg5174Isup2.hkl

Supplementary material file. DOI: 10.1107/S1600536812005119/hg5174Isup3.cml

Additional supplementary materials:  crystallographic information; 3D view; checkCIF report

## Figures and Tables

**Table 1:** Hydrogen-bond geometry (Å, °)

*D*—H⋯*A*	*D*—H	H⋯*A*	*D*⋯*A*	*D*—H⋯*A*
N1—H1N⋯S1^i^	0.87 (1)	2.51 (1)	3.3723 (11)	172 (2)
N2—H2N⋯O1^ii^	0.87 (1)	1.96 (1)	2.8210 (14)	168 (2)

Symmetry codes: (i) 
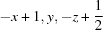
; (ii) 
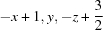
.

**Fig. 1.**
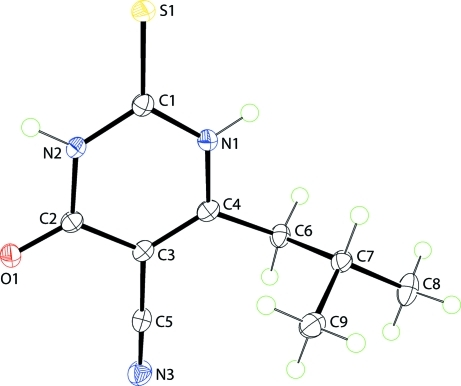
The molecular structure of (I) showing the atom-labelling scheme and displacement ellipsoids at the 50% probability level.

**Fig. 2.**
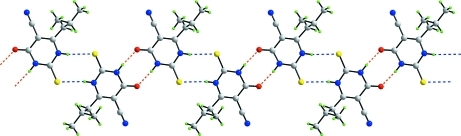
A view of the linear supramolecular chain along [001] in (I). The N—H···O and N—H···S hydrogen bonds are shown as orange and blue dashed lines, respectively.

**Fig. 3.**
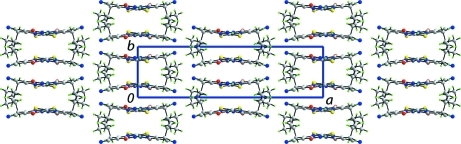
A view in projection down the c axis of the unit-cell contents for (I). The N—H···O and N—H···S interactions are shown as orange and blue dashed lines, respectively.

## supplementary crystallographic information

### Comment

The chemotherapeutic efficacy of uracil derivatives is related to their ability to inhibit vital enzymes responsible for DNA biosynthesis. Thus, several uracil and pyrimidine non-nucleoside derivatives exhibited anti-cancer (Al-Safarjalani *et al.*, 2005), anti-viral (Brunelle *et al.*, 2007; Ding *et al.*, 2006) and anti-bacterial activities (Hawser *et al.*, 2006; Al-Abdullah *et al.*, 2011). In continuation to our interest in the chemical and pharmacological properties of uracil and pyrimidine derivatives (Al-Omar *et al.*, 2010; Al-Turkistani *et al.*, 2011), and as part of on-going structural studies of uracil and pyrimidine derivatives (Nasir *et al.*, 2010; El-Emam *et al.* 2011), we synthesized the title compound 6-(2-methylpropyl)-2-thiouracil-5-carbonitrile (I) as a precursor for potential chemotherapeutic agents.

The thiouracil derivative (I), Fig. 1, exists in the thione form with the C1═S1 bond length of 1.6693 (13) Å being shorter than the equivalent bond in the parent 2-thiouracil compound, *i.e.* 1.683 (3) Å (Tiekink, 1989). The six atoms comprising the ring are co-planar, having a r.m.s. deviation = 0.015 Å. The 2-methylpropyl group lies to one side of the central plane with the N1—C4—C6—C7 torsion angle being 72.88 (14)°.

The crystal packing features N—H···O and N—H···S hydrogen bonding involving both amide-H and the oxo and thione atoms, Table 1. The result of these hydrogen bonds is the formation of linear supramolecular chains along the *c* axis featuring alternating eight-membered {···HNCO}_2_ and {···HNCS}_2_ synthons, Fig. 2. The chains stack in the crystal structure with no specific intermolecular interactions between them, Fig. 3.

### Experimental

A mixture of 3-methylbutanal (8.61 g, 0.1 mol), ethyl cyanoacetate (11.31 g, 0.1 mol), thiourea (7.61 g, 0.1 mol) and potassium carbonate (13.8 g, 0.1 mol) was heated in in ethanol (300 ml) under reflux for 6 h. On cooling, the separated precipitate was filtered, washed with diethyl ether and dried. The obtained solid was added to water (200 ml) and the mixture was heated at 283–293 K until a clear solution was obtained. The solution was acidified with acetic acid and stirred for 30 min. The deposited precipitate was filtered, washed with cold water, dried and crystallized from acetic acid to yield 5.86 g (28%) of the title compound (I) as colourless crystals. m.p. 545–547. ^1^H NMR (DMSO-d6): δ 1.07 (d, 6H, CH_3_, J = 6.5 Hz), 2.18–2.24 (m, 1H, CH), 2.68 (d, 2H, CH_2_, J = 6.5 Hz), 13.08 (br. s, 2H, NH). ^13^C NMR: δ 21.50 (CH_3_), 29.40 (CH), 40.30 (CH_2_), 92.30 (Uracil C-5), 114.80 (CN), 158.60 (C═O), 163.90 (Uracil C-6), 176.75 (C═S).

### Refinement

Carbon-bound H atoms were placed in calculated positions [C—H = 0.95 to 1.00 Å, *U*_iso_(H) = 1.2–1.5*U*_eq_(C)] and were included in the refinement in the riding model approximation. The amide H atoms were located in a difference Fourier map, and were refined with a distance restraint of N—H = 0.88±0.01 Å; their *U*_iso_ values were refined.

### Crystal data

**Table:**

C_9_H_11_N_3_OS	*F*(000) = 880
*M**_r_* = 209.27	*D*_x_ = 1.377 Mg m^−^^3^
Monoclinic, *C*2/*c*	Cu *K*α radiation, λ = 1.54184 Å
Hall symbol: -C 2yc	Cell parameters from 4317 reflections
*a* = 25.8985 (6) Å	θ = 4.0–76.1°
*b* = 7.0479 (2) Å	µ = 2.62 mm^−^^1^
*c* = 11.1811 (2) Å	*T* = 100 K
β = 98.527 (2)°	Needle, colourless
*V* = 2018.33 (8) Å^3^	0.35 × 0.20 × 0.03 mm
*Z* = 8	

### Data collection

**Table:**

Agilent SuperNova Dual diffractometer with Atlas detector	2102 independent reflections
Radiation source: SuperNova (Cu) X-ray Source	1989 reflections with *I* > 2σ(*I*)
Mirror monochromator	*R*_int_ = 0.024
Detector resolution: 10.4041 pixels mm^-1^	θ_max_ = 76.3°, θ_min_ = 6.5°
ω scans	*h* = −31→32
Absorption correction: multi-scan (*CrysAlis PRO*; Agilent, 2011)	*k* = −8→8
*T*_min_ = 0.461, *T*_max_ = 0.926	*l* = −14→10
6870 measured reflections	

### Refinement

**Table:**

Refinement on *F*^2^	Primary atom site location: structure-invariant direct methods
Least-squares matrix: full	Secondary atom site location: difference Fourier map
*R*[*F*^2^ > 2σ(*F*^2^)] = 0.030	Hydrogen site location: inferred from neighbouring sites
*wR*(*F*^2^) = 0.086	H atoms treated by a mixture of independent and constrained refinement
*S* = 1.03	*w* = 1/[σ^2^(*F*_o_^2^) + (0.0528*P*)^2^ + 1.3984*P*] where *P* = (*F*_o_^2^ + 2*F*_c_^2^)/3
2102 reflections	(Δ/σ)_max_ = 0.002
135 parameters	Δρ_max_ = 0.27 e Å^−^^3^
2 restraints	Δρ_min_ = −0.28 e Å^−^^3^

### Special details

**Table:**

Geometry. All e.s.d.'s (except the e.s.d. in the dihedral angle between two l.s. planes) are estimated using the full covariance matrix. The cell e.s.d.'s are taken into account individually in the estimation of e.s.d.'s in distances, angles and torsion angles; correlations between e.s.d.'s in cell parameters are only used when they are defined by crystal symmetry. An approximate (isotropic) treatment of cell e.s.d.'s is used for estimating e.s.d.'s involving l.s. planes.
Refinement. Refinement of *F*^2^ against ALL reflections. The weighted *R*-factor *wR* and goodness of fit *S* are based on *F*^2^, conventional *R*-factors *R* are based on *F*, with *F* set to zero for negative *F*^2^. The threshold expression of *F*^2^ > σ(*F*^2^) is used only for calculating *R*-factors(gt) *etc*. and is not relevant to the choice of reflections for refinement. *R*-factors based on *F*^2^ are statistically about twice as large as those based on *F*, and *R*-factors based on ALL data will be even larger.

### Fractional atomic coordinates and isotropic or equivalent isotropic displacement parameters (Å^2^)

**Table:**

	*x*	*y*	*z*	*U*_iso_*/*U*_eq_	
S1	0.550100 (11)	0.21184 (4)	0.42164 (3)	0.01651 (12)	
O1	0.43446 (3)	0.31829 (14)	0.72665 (8)	0.0189 (2)	
N1	0.44705 (4)	0.25643 (16)	0.37760 (9)	0.0154 (2)	
N2	0.48422 (4)	0.27666 (15)	0.57730 (9)	0.0143 (2)	
N3	0.30186 (4)	0.37646 (18)	0.58639 (10)	0.0225 (3)	
C1	0.49111 (5)	0.24972 (18)	0.46022 (11)	0.0144 (2)	
C2	0.43658 (5)	0.30269 (17)	0.61833 (11)	0.0146 (3)	
C3	0.39202 (5)	0.31030 (17)	0.52342 (11)	0.0150 (3)	
C4	0.39788 (5)	0.28817 (16)	0.40487 (11)	0.0151 (3)	
C5	0.34180 (5)	0.34614 (19)	0.55782 (10)	0.0166 (3)	
C6	0.35440 (5)	0.29348 (18)	0.30154 (11)	0.0165 (3)	
H6A	0.3644	0.3779	0.2381	0.020*	
H6B	0.3232	0.3490	0.3297	0.020*	
C7	0.33981 (5)	0.09633 (18)	0.24532 (10)	0.0160 (3)	
H7	0.3698	0.0480	0.2073	0.019*	
C8	0.29278 (5)	0.1211 (2)	0.14684 (12)	0.0246 (3)	
H8A	0.3012	0.2133	0.0870	0.037*	
H8B	0.2629	0.1670	0.1831	0.037*	
H8C	0.2840	−0.0010	0.1071	0.037*	
C9	0.32809 (5)	−0.0459 (2)	0.34028 (12)	0.0221 (3)	
H9A	0.3589	−0.0599	0.4022	0.033*	
H9B	0.3193	−0.1689	0.3017	0.033*	
H9C	0.2986	−0.0004	0.3780	0.033*	
H1N	0.4514 (7)	0.243 (3)	0.3021 (9)	0.026 (4)*	
H2N	0.5113 (5)	0.275 (3)	0.6341 (13)	0.023 (4)*	

### Atomic displacement parameters (Å^2^)

**Table:**

	*U*^11^	*U*^22^	*U*^33^	*U*^12^	*U*^13^	*U*^23^
S1	0.01253 (17)	0.02143 (19)	0.01565 (18)	0.00095 (10)	0.00231 (11)	0.00078 (10)
O1	0.0159 (4)	0.0270 (5)	0.0136 (4)	0.0004 (4)	0.0013 (3)	−0.0010 (3)
N1	0.0141 (5)	0.0193 (5)	0.0125 (5)	−0.0002 (4)	0.0014 (4)	−0.0001 (4)
N2	0.0112 (5)	0.0176 (5)	0.0134 (5)	0.0001 (4)	−0.0003 (4)	−0.0001 (4)
N3	0.0173 (5)	0.0264 (6)	0.0232 (5)	0.0018 (5)	0.0018 (4)	−0.0033 (5)
C1	0.0153 (6)	0.0121 (5)	0.0155 (6)	−0.0014 (4)	0.0009 (4)	0.0007 (4)
C2	0.0137 (6)	0.0130 (6)	0.0168 (6)	−0.0004 (4)	0.0016 (5)	0.0000 (4)
C3	0.0128 (6)	0.0149 (6)	0.0167 (6)	−0.0003 (4)	0.0006 (4)	−0.0003 (4)
C4	0.0139 (6)	0.0122 (6)	0.0186 (6)	−0.0003 (4)	0.0009 (5)	0.0006 (4)
C5	0.0165 (6)	0.0168 (6)	0.0152 (5)	0.0002 (5)	−0.0014 (4)	−0.0016 (4)
C6	0.0150 (6)	0.0183 (7)	0.0152 (6)	0.0013 (4)	−0.0012 (5)	−0.0003 (4)
C7	0.0122 (5)	0.0193 (6)	0.0162 (5)	0.0005 (4)	0.0011 (4)	−0.0028 (5)
C8	0.0230 (7)	0.0251 (7)	0.0224 (6)	0.0030 (5)	−0.0075 (5)	−0.0055 (5)
C9	0.0201 (6)	0.0233 (7)	0.0224 (6)	−0.0050 (5)	0.0014 (5)	0.0004 (5)

### Geometric parameters (Å, º)

**Table:**

S1—C1	1.6693 (13)	C6—C7	1.5486 (17)
O1—C2	1.2254 (15)	C6—H6A	0.9900
N1—C1	1.3583 (16)	C6—H6B	0.9900
N1—C4	1.3711 (16)	C7—C9	1.5232 (18)
N1—H1N	0.873 (9)	C7—C8	1.5261 (16)
N2—C1	1.3605 (16)	C7—H7	1.0000
N2—C2	1.3909 (16)	C8—H8A	0.9800
N2—H2N	0.874 (9)	C8—H8B	0.9800
N3—C5	1.1470 (17)	C8—H8C	0.9800
C2—C3	1.4482 (17)	C9—H9A	0.9800
C3—C4	1.3653 (18)	C9—H9B	0.9800
C3—C5	1.4323 (17)	C9—H9C	0.9800
C4—C6	1.4895 (17)		
			
C1—N1—C4	124.66 (11)	C4—C6—H6B	108.8
C1—N1—H1N	116.1 (12)	C7—C6—H6B	108.8
C4—N1—H1N	119.2 (12)	H6A—C6—H6B	107.7
C1—N2—C2	125.81 (10)	C9—C7—C8	110.98 (10)
C1—N2—H2N	119.4 (12)	C9—C7—C6	111.67 (10)
C2—N2—H2N	114.7 (12)	C8—C7—C6	108.10 (10)
N1—C1—N2	115.64 (11)	C9—C7—H7	108.7
N1—C1—S1	122.58 (9)	C8—C7—H7	108.7
N2—C1—S1	121.77 (9)	C6—C7—H7	108.7
O1—C2—N2	120.65 (11)	C7—C8—H8A	109.5
O1—C2—C3	124.98 (11)	C7—C8—H8B	109.5
N2—C2—C3	114.37 (10)	H8A—C8—H8B	109.5
C4—C3—C5	121.13 (11)	C7—C8—H8C	109.5
C4—C3—C2	121.06 (11)	H8A—C8—H8C	109.5
C5—C3—C2	117.79 (11)	H8B—C8—H8C	109.5
N1—C4—C3	118.37 (11)	C7—C9—H9A	109.5
N1—C4—C6	116.87 (11)	C7—C9—H9B	109.5
C3—C4—C6	124.75 (12)	H9A—C9—H9B	109.5
N3—C5—C3	179.16 (14)	C7—C9—H9C	109.5
C4—C6—C7	113.78 (10)	H9A—C9—H9C	109.5
C4—C6—H6A	108.8	H9B—C9—H9C	109.5
C7—C6—H6A	108.8		
			
C4—N1—C1—N2	0.16 (19)	C1—N1—C4—C3	−1.67 (19)
C4—N1—C1—S1	−179.61 (10)	C1—N1—C4—C6	179.07 (12)
C2—N2—C1—N1	2.54 (19)	C5—C3—C4—N1	179.23 (11)
C2—N2—C1—S1	−177.70 (9)	C2—C3—C4—N1	0.68 (18)
C1—N2—C2—O1	177.06 (12)	C5—C3—C4—C6	−1.58 (19)
C1—N2—C2—C3	−3.35 (18)	C2—C3—C4—C6	179.87 (11)
O1—C2—C3—C4	−178.83 (12)	N1—C4—C6—C7	72.88 (14)
N2—C2—C3—C4	1.60 (17)	C3—C4—C6—C7	−106.32 (14)
O1—C2—C3—C5	2.58 (19)	C4—C6—C7—C9	53.98 (14)
N2—C2—C3—C5	−176.99 (11)	C4—C6—C7—C8	176.33 (11)

### Hydrogen-bond geometry (Å, º)

**Table:**

*D*—H···*A*	*D*—H	H···*A*	*D*···*A*	*D*—H···*A*
N1—H1*N*···S1^i^	0.87 (1)	2.51 (1)	3.3723 (11)	172 (2)
N2—H2*N*···O1^ii^	0.87 (1)	1.96 (1)	2.8210 (14)	168 (2)

Symmetry codes: (i) −*x*+1, *y*, −*z*+1/2; (ii) −*x*+1, *y*, −*z*+3/2.
